# Prevalence and adverse outcomes of frailty in older patients with acute myocardial infarction after percutaneous coronary interventions: A systematic review and meta‐analysis

**DOI:** 10.1002/clc.23929

**Published:** 2022-09-28

**Authors:** Qian Yu, Dawei Guo, Jianan Peng, Qifei Wu, Yonghuan Yao, Mei Ding, Jiang Wang

**Affiliations:** ^1^ College of Nursing Gannan Medical University Ganzhou Jiangxi China; ^2^ Department of Medicine JingGangshan University Ji'an Jiangxi China

**Keywords:** acute myocardial infarction, frailty, outcome, percutaneous coronary intervention, prevalence

## Abstract

**Background:**

The association between frailty and older patients with acute myocardial infarction (AMI) after percutaneous coronary intervention (PCI) is unclear. Therefore, we conducted a systematic review and meta‐analysis to investigate the prevalence of frailty in older patients with AMI following PCI, and determine the relationship between frailty and adverse outcomes in these patients.

**Hypothesis:**

Older patients with AMI have a higher prevalence of frailty after PCI, and the frailty in these patients increases the risk of adverse outcomes.

**Methods:**

A comprehensive search of the PubMed, Cochrane, Ovid (Medline), Ovid (Embase), and Web of Science databases was performed for articles published until October 2021. A meta‐analysis was performed using stata12.0 software. A random‐effects model was used when *I* ^2^ was greater than 50%; otherwise, a fixed‐effects model was used.

**Results:**

There were a total of 274,976 older patients in the included studies. Nine studies investigated the prevalence of frailty in older patients with AMI after PCI, with an overall prevalence of 39% (95% confidence interval [CI]: 18%–60%, *p* < .001). Six studies included adverse outcomes of frailty in older patients with AMI after PCI, including all‐cause mortality (hazard ratio [HR] = 2.29, 95% CI: 1.65–3.16, *p* = .285), rehospitalization (HR = 2.53, 95% CI: 1.38–4.63), and in‐hospital major bleeding (HR = 1.93, 95% CI: 1.29–2.90, *p* = .825).

**Conclusion:**

The frailty prevalence is increased in older patients with AMI after PCI, especially in ST‐segment elevation myocardial infarction (STEMI). AMI with frailty after PCI is more likely to be associated with worse clinical outcomes, such as death, bleeding, and rehospitalization.

## 1 INTRODUCTION

Acute myocardial infarction (AMI) is a common cardiovascular disease, usually linked to myocardial cell necrosis caused by prolonged ischemia and hypoxia of the heart.^1^ In recent years, the implementation of percutaneous coronary intervention (PCI) has become more frequent, and the adverse clinical outcomes of patients with AMI have improved.^2^

Frailty is a common geriatric symptom that is more common in cardiovascular disease than in the general population,^3^ the clinical manifestations of which include weight loss, increased fatigue, diminished grip strength, decreased walking speed, and reduced activity.^4^ In addition, frailty affects the quality of life of older population and can increase the risk of falls, hospitalization, and death.^5^ Studies have shown that advancing age also increases the prevalence of frailty, with an increase of 10% over 65 years and 30% over 80 years.^6^

Older adults with cardiovascular disease are three times more likely to suffer from frailty and two times as likely to die as healthy older adults.^7^ Furthermore, the risk of frailty during hospitalization in patients with AMI increases due to the burden of disease and prolonged bed rest.^2^ Studies have shown that frailty can predict the risk of adverse outcomes in cardiovascular disease.^7^, ^8^, ^9^

However, the prevalence of frailty in older patients with AMI after PCI and the risk of adverse outcomes after discharge remain unclear. Therefore, we conducted a systematic review and meta‐analysis to assess the incidence of frailty and its adverse clinical effects in older patients with AMI after PCI.

## 2 METHODS

### 2.1 Search strategy and study selection

The PubMed, Cochrane, Ovid (Medline), Ovid (Embase), and Web of Science databases were searched for relevant literature published in English from the establishment of each database to October 2021. The search strategy for PubMed was as follows: frailty [MeSH terms] OR frailty * [title/abstract] AND myocardial infarction [MeSH terms] OR ((myocardial infarct* [title/abstract]) OR (heart attack* [title/abstract]) OR (attack* [title/abstract]) OR (AMI [title/abstract]). References from the included articles published in English were manually searched.

### 2.2 Study inclusion and exclusion criteria

We included the following studies: (1) studies in which frailty was limited to a severely frail population; (2) cross‐sectional or cohort studies which reported the prevalence of frailty in older patients with AMI after PCI; (3) studies in which the primary clinical outcomes included all‐cause, in‐hospital, and midterm deaths, and the secondary clinical outcomes were bleeding or rehospitalization, referring to intracranial, abdominal, and pelvic bleeding due to any cause; and (4) articles in English.

Two independent evaluators (Qifei Wu and Yonghuan Yao) performed literature screening to determine whether the studies met the inclusion criteria. In case of a disagreement, a third evaluator (Qian Yu) was consulted.

### 2.3 Data extraction

Two investigators (Qifei Wu and Yonghuan Yao) extracted the data required for this study in an Excel spreadsheet according to the purpose of the study. The data extracted included: (1) study type and country, (2) disease type, (3) sample number, age, and percentage of males, (4) frailty assessment tools, (5) frailty prevalence, (6) adverse outcomes, and (7) follow‐up time. To ensure the accuracy of data extraction, two investigators separately performed the data extraction process. Any disagreement during the data extraction was resolved by a focused discussion or by clarification with a third investigator (Qian Yu).

### 2.4 Evaluation of literature quality

Two investigators (Dawei Guo Jianan Peng) independently evaluated the quality of the included literature using the Newcastle–Ottawa Quality Scale.^10^ This scale contains eight items in three dimensions: study population selection, comparability, and outcome measurement. The total score was 9 points. The quality scores of the studies included in this meta‐analysis were between 7 and 9 points. Four of the included studies had full scores, indicating that they had high quality.

### 2.5 Statistical analysis

Statistical analysis was performed using Stata 12.0. The *Q*‐test was used to analyze heterogeneity. Heterogeneity was considered significant at *p* ≤ .10. Heterogeneity was evaluated by combining this with the *I* ^2^ statistic. The larger the *I* ^2^ statistic, the more important the heterogeneity.^11^ When *I* ^2^ ≤ 50%, the fixed‐effect model was used; if *I* ^2^ > 50%, the subgroup analysis and the random‐effects model were used to explore the sources of heterogeneity. For clinical outcomes, we performed a subgroup analysis to calculate the relative risk and 95% confidence interval (CI) of the association between frailty and AMI after PCI and all‐cause mortality, in‐hospital major bleeding, and rehospitalization. Sensitivity analysis was performed to verify whether the results were stable and reliable, and funnel plots were used to assess whether there was publication bias.^12^ We set the funnel plot asymmetry and defined significant publication bias as a *p*‐value < .05 using the Begg and Egger test.

## 3 RESULTS

### 3.1 Literature search and screening results

A total of 2953 articles were obtained through database searches, comprising 402 articles from PubMed, 89 articles from Cochrane, 1238 articles from Ovid (Embase), 371 articles from Ovid (Medline), and 853 articles from Web of Science. After excluding duplicate publications and articles unrelated to the topic or abstract, 51 articles remained; 41 of which were excluded because of the study type (review, comment or letter, randomized controlled trial), or because data showing the prevalence of frailty or adverse outcomes were lacking or not associated with the primary clinical outcome. Eventually, 10 studies on the prevalence of frailty and the correlation of adverse outcomes in older patients with AMI after PCI were included (Supporting Information: Figure 1).

### 3.2 Basic characteristics of the included studies

Information from 10 studies including a total of 274,976 older patients with AMI after PCI was included in our study (Table 1). These 10 trials were all published in 2018–2021, with the majority (6) published in 2019,^2^, ^3^, ^13^, ^14^, ^15^, ^16^, ^17^, ^18^, ^19^, ^20^ and had either a cohort or cross‐sectional study design. Frailty assessment tools mainly included the frailty point scoring system (FPSS), frailty index, and clinical frailty scale. This study aimed to evaluate frailty in older patients with AMI after PCI. The primary adverse outcomes were all‐cause mortality, rehospitalization, and bleeding during follow‐up, with a maximum follow‐up of 2 years in the included studies.^17^ Overall, nine studies investigated the prevalence of frailty in older patients with AMI after PCI,^3^, ^13^, ^14^, ^15^, ^16^, ^17^, ^18^, ^19^, ^20^ and six involved adverse outcomes in this population.^2^, ^16^, ^17^, ^18^, ^19^, ^20^

**Table 1:** Baseline characteristics and summary of included studies

Author year	Study type	Country	Disease type	*N*	Age	Male (%)	Frailty assessment tool	Prevalence (%)	Outcome	Follow‐up time	Quality score
Damluji, 2019^3^	Cross‐sectional study, cohort study	United States	AMI undergone PCI	140,089	≥75 years	46.8	FI	9.9	NA	NA	8
Uchmano‐wicz, 2019^13^	Cross‐sectional study	Poland	NSTEMI undergone PCI	100	66.12 ± 10.92	61.0	TFI	80.0	NA	NA	7
Dodson, 2018,^14^	Cross‐sectional study	United States	NSTEMI undergone PCI	129,300	75.3 ± 7.7	60.2	FPSS	19.8	NA	NA	9
			STEMI undergone PCI					78.2			
Patel, 2018,^15^	Cross‐sectional study, cohort study	Australia	STEMI undergone PCI	1275	≥65 years	NA	FI	52.6	NA	NA	9
			NSTEMI undergone PCI	2669				25.3			
Nishihira, 2020,^2^	Cohort study	Japan	AMI undergone PCI	96	92 (91–94)	36.0	FPSS	NA	In‐hospital major bleeding	375 days	7
									Midterm mortality		
Nguyen, 2019,^16^	Cross‐sectional study, cohort study	Vietnam	AMI undergone PCI	163	73.5 ± 8.3	60.8	REFS	41.7	30‐day mortality	30 days	8
									30‐day readmission		
Yoshioka, 2019^17^	Cross‐sectional study, cohort study	Japan	STEMI undergone PCI	273	84.6 ± 3.8	46.2	CSHA‐CFS	48.7	All‐cause mortality	2 years	9
Hermans, 2019^18^	Cohort study	Netherlands	STEMI undergone PCI	206	79 ± 6.4	58.0	VMS score	27.7	All‐cause mortality	30 days	8
Nishihira, 2021,^19^	Prospective study	NA	AMI undergone PCI	546	84.5 (82–88)	47.8	FPSS	27.8	All‐cause mortality	589 days	9
									In‐hospital major bleeding		
Calvo, 2019^20^	Observational prospective study	NA	STEMI undergone PCI	259	82.6 ± 6	57.9	FS	19.7	In‐hospital mortality	NA	7

Abbreviations: AMI, acute myocardial infarction; CSHA‐CFS, The Canadian study of health and aging clinical frailty scale; FI, frailty index; FPSS, frailty point scoring system; FS, FRAIL scale; NA, not available; NSTEMI, non‐ST elevation myocardial infarction; PCI, percutaneous coronary intervention; REFS, Reported Edmonton Frail Scale; STEMI: ST‐elevation myocardial infarction; TFI, Tilburg Frailty Indicator; VMS score, Veiligheids Management System.

### 3.3 Meta‐analysis results

**Figure 1:**
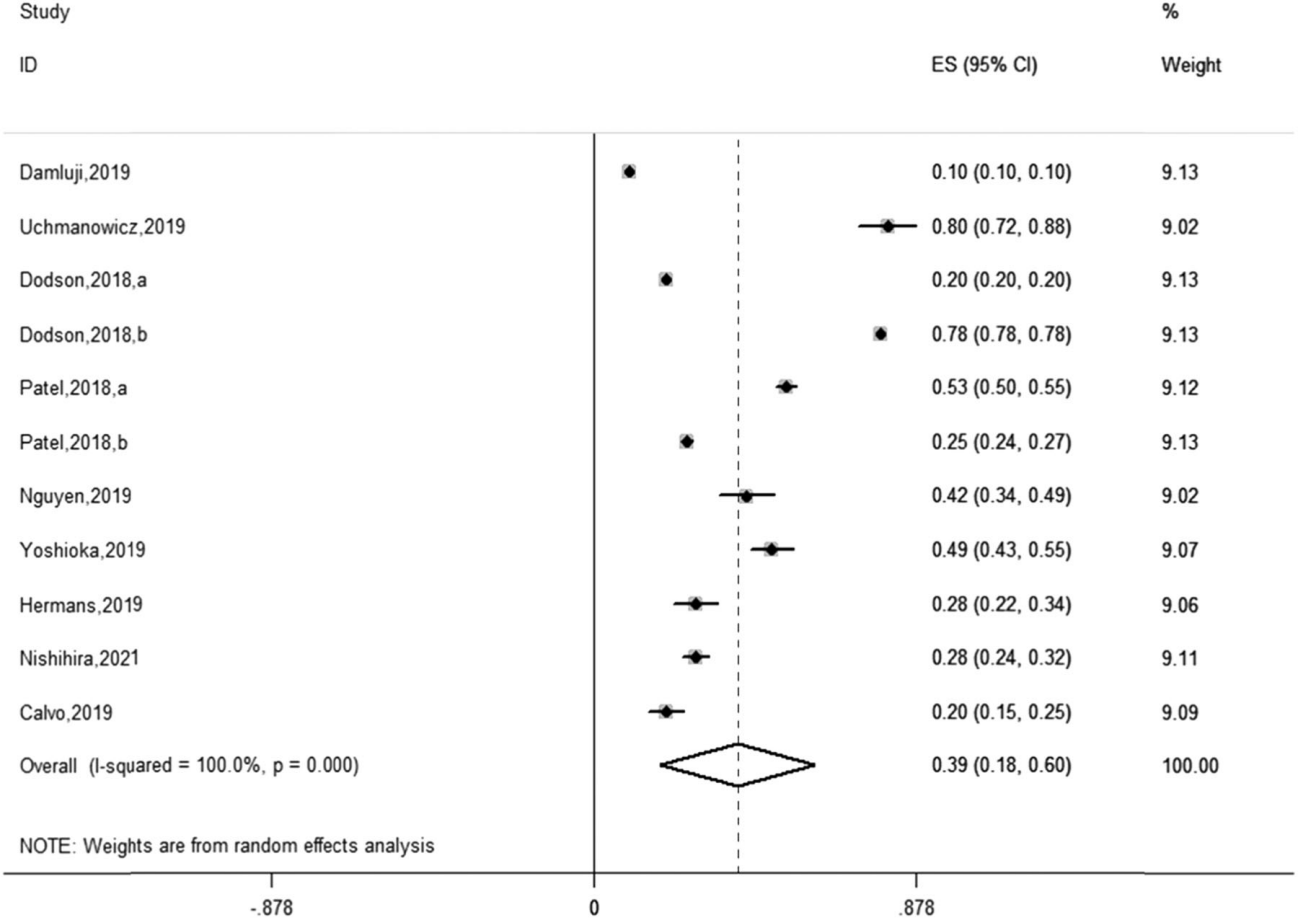
Forest plot showing the prevalence of frail patients with older AMI after PCI. AMI, acute myocardial infarction; PCI, percutaneous coronary intervention.

**Figure 2:**
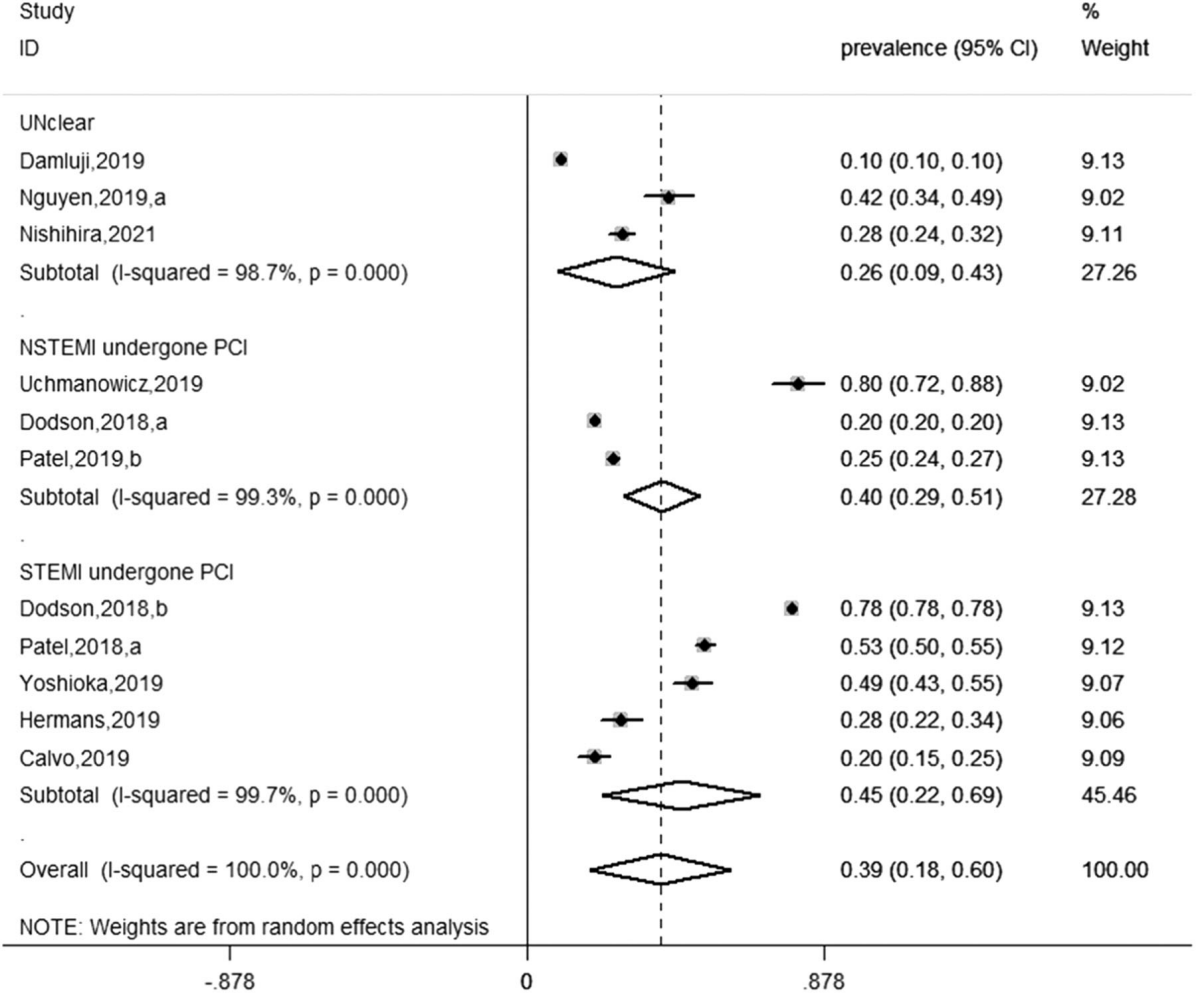
Forest plot showing subgroup analysis of the type of older AMI after PCI. AMI, acute myocardial infarction; PCI, percutaneous coronary intervention.

Figure 1 shows a meta‐analysis of the prevalence of frail older patients with AMI after PCI. Of the 10 studies included from 2018 to 2021, 9 included data on the frailty prevalence of 274,880 older patients with AMI after PCI. The overall prevalence of AMI in older patients after PCI was 39% (95% CI: 18%–60%) in the heterogeneity test (*I* ^2^ = 100%, *p* < .001). Our study suggested significant differences in the prevalence of AMI frailty in older patients after PCI. We performed a subgroup analysis of the type of AMI in older patients (Figure 2). Disease types are mainly divided into non‐ST‐segment elevation myocardial infarction (NSTEMI) patients underwent PCI, STEMI patients underwent PCI, and other types were uniformly classified as unclear. Among them, the studies by Dodson et al.^14^ and Patel et al.^15^ included older patients with STEMI and NSTEMI who underwent PCI. The prevalence of NSTEMI in patients who developed frailty following PCI was 40% (95% CI: 29%–51%) in the heterogeneity test (*I* ^2^ = 99.3%, *p* < .001), and that of STEMI was 45% (95% CI: 22%–69%) in the heterogeneity test (*I* ^2^ = 99.7%, *p* < .001).

**Figure 3:**
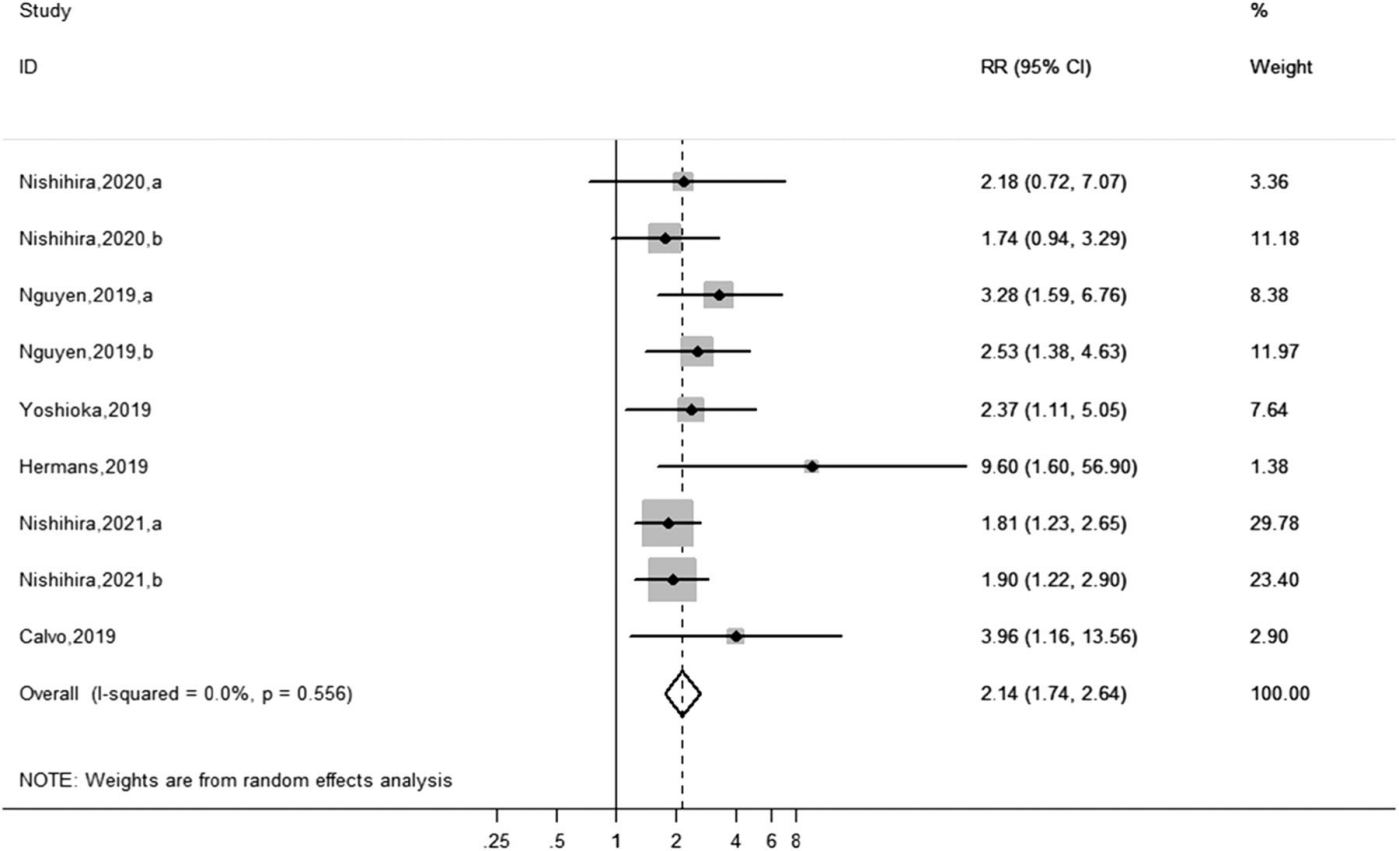
Forest plot showing risk of adverse outcomes of frail participants with older AMI after PCI. AMI, acute myocardial infarction; PCI, percutaneous coronary intervention.

**Figure 4:**
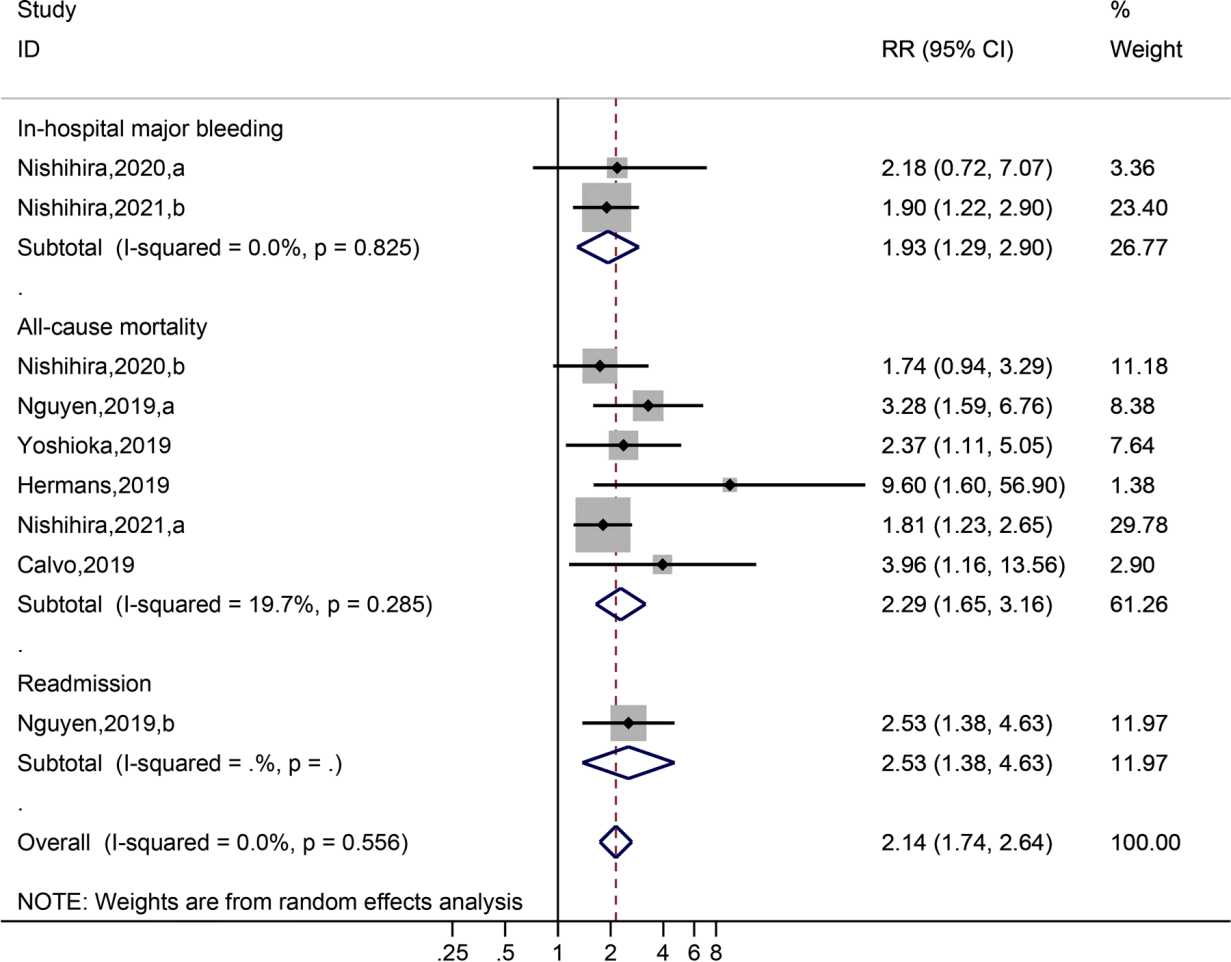
Forest plot showing subgroup analysis of adverse outcomes of frail participants with older AMI after PCI. AMI, acute myocardial infarction; PCI, percutaneous coronary intervention.

Older patients with frailty who underwent AMI after PCI had a markedly increased risk of adverse outcomes compared with non‐frail patients with AMI after PCI (HR = 2.14, 95% CI: 1.74–2.64, *p* = .556, *I* ^2^ = 0.0%). A forest plot for this meta‐analysis is shown in Figure 3. We performed subgroup analysis of the types of adverse outcomes (Figure 4), including six studies comprising 1543 older adults with adverse clinical outcomes. The results showed that all‐cause mortality was significantly increased in frail older patients with AMI after PCI than in non‐frail older patients with AMI after PCI (HR = 2.29, 95% CI: 1.65–3.16, *p* = .285, *I* ^2^ = 19.7%). In studies published by Nishihira et al. in 2020 and 2021,^2^, ^19^ adverse outcomes involved in‐hospital bleeding, and the risk of bleeding was significantly increased in frail older patients with AMI after PCI than in non‐frail older patients with AMI after PCI (HR = 1.93, 95% CI: 1.29–2.90). Nguyen et al. studied all‐cause mortality and rehospitalization.^16^ After PCI, frail older patients with AMI had an increased risk of rehospitalization (HR = 2.53, 95% CI: 1.38–4.63). Other subgroup analyses of the prevalence of AMI after PCI in frail older participants are shown in Supporting Information: Table 1, while other subgroup analyses of adverse outcomes in frail older participants with AMI after PCI are shown in Supporting Information: Table 2.

## 4 DISCUSSION

This study summarizes the relationship between the prevalence of frailty and adverse outcomes in older patients with AMI after PCI. Approximately 39% of older patients with AMI develop frailty after PCI, which is more significant in older patients with STEMI undergoing PCI. Among the adverse outcomes, we found that frail older patients with AMI undergoing PCI had approximately twice the risk of all‐cause mortality, rehospitalization, and bleeding as in the non‐frail population.

PCI is the most common revascularization method for cardiovascular diseases and can significantly improve cardiovascular problems. Studies have shown that older AMI patients have an increased risk of requiring PCI and an increased prevalence of frailty.^3^ The definition of frailty and assessment tools varies across studies, which consequently results in variations in the reported prevalence.^21^

In this meta‐analysis, the most common frailty assessment tool was the FPSS. FPSS is a new concept about frailty based on Canadian health and aging research by Dodson et al.^14^ and includes three variables: walking, cognitive function, and basic daily life. The number of items is small, the clinical application is relatively convenient, and different degrees of frailty can also be well assessed in the acute onset of AMI. The second is the frailty index, which is based on the cumulative deficit model. The frailty index takes the human body as a unified whole, and when the cumulative deficits reach a certain level, older patients with AMI will have a poor prognosis.^15^ Finally, the Canadian Clinical Frailty Scale for Health and Aging (CFS), a rating score scale developed by RockWood et al.^22^ A higher score on this scale indicates more severe frailty, and studies have shown that the CFS can predict the risk of STEMI death after PCI.^17^

With improvements in medical technology, myocardial reperfusion therapy, PCI, thrombolytic therapy, and secondary drug prevention, the mortality rate of STEMI has decreased.^23^ Sujino et al. showed that early PCI surgery in older patients may contribute to physical recovery, while PCI may have poor adverse clinical outcomes in patients with severe frailty.^24^ At the same time, according to the research report of Nishihira et al., frailty is associated with a higher risk of death.^19^ Qin et al. showed that patients aged ≥75 years with AMI had a higher risk of readmission 30 days after PCI, and the reasons for readmission were heart failure and infection.^25^ In addition, In the acute phase, older patients may bleed during treatment. Bleeding reduces the quality of life of older patients after discharge, and patients with bleeding are more likely to experience complications outside the hospital (such as falls, disability, rehospitalization, and even death), which are considered to be related to frailty.^14^, ^26^, ^27^, ^28^

Now, in the United States, more than 1 million adults suffer from myocardial infarction each year.^29^ Therefore, early intervention in patients with AMI can improve the prognosis of patients. The American Heart Association/American College of Cardiology recommends cardiac rehabilitation (CR) for secondary prevention in patients with cardiovascular disease.^30^ CR refers to rehabilitation treatment that allows patients with heart disease to live autonomously through specific intervention methods.^31^ In 2017, the European Society of Cardiology indicated that all patients with AMI should actively participate in cardiac rehabilitation programs,^32^ consisting of appropriate exercise training, lifestyle changes, and weight and nutritional management.^31^ Participation in cardiac rehabilitation programs can help patients delay the frailty process and reduce the likelihood of falls and rehospitalizations after discharge. This enhances cardiac tolerance and reduces mortality, and helps the patient regain maximum strength and a healthy life.^32^, ^33^, ^34^

## 5 CONCLUSION

Overall, our analysis showed that the prevalence of frailty increases in older patients with AMI undergoing PCI, especially in patients with STEMI. Frail older patients with AMI undergoing PCI are more likely to experience worse clinical outcomes, such as death, bleeding, and rehospitalization.

## 6 LIMITATION

This study has several limitations. On the one hand, there was heterogeneity due to inconsistent definitions of frailty in different studies, the different frailty assessment tools used, and the different degrees of measured frailty. However, we did not perform publication bias detection because of the limited number of samples with adverse outcomes.

## AUTHOR CONTRIBUTIONS

Mei Ding and Jiang Wang conceived and designed the study. Qifei Wu and Yonghuan Yao performed the literature search and the data extraction. Dawei Guo and Jianan Peng participated in the methodological quality assessment and in the statistical analysis. The protocol manuscripts were finished by Jianan Peng. All authors have checked manuscripts and approved the publication of the protocol.

## CONFLICT OF INTEREST

The authors declare no conflict of interest.

## Supporting information

Supplementary information.

Supplementary information.

Supplementary information.

## Data Availability

All data generated or analyzed during this study are included in this published article.

## References

[1] Anderson JL , Morrow DA . Acute myocardial infarction. N Engl J Med. 2017;376(21):2053‐2064. 28538121 10.1056/NEJMra1606915

[2] Nishihira K , Watanabe N , Kuriyama N , Shibata Y . Clinical outcomes of nonagenarians with acute myocardial infarction who undergo percutaneous coronary intervention. Eur Heart J Acute Cardiovasc Care. 2020;9(5):488‐495. 32324045 10.1177/2048872620921596

[3] Damluji AA , Huang J , Bandeen‐Roche K , et al. Frailty among older adults with acute myocardial infarction and outcomes from percutaneous coronary interventions. J Am Heart Assoc. 2019;8(17):e013686. 31475601 10.1161/JAHA.119.013686 PMC6755849

[4] Fried LP , Tangen CM , Walston J , et al. Frailty in older adults: evidence for a phenotype. J Gerontol A Biol Sci Med Sci. 2001;56(3):M146‐M156. 11253156 10.1093/gerona/56.3.m146

[5] Struijk EA , Hagan KA , Fung TT , Hu FB , Rodríguez‐Artalejo F , Lopez‐Garcia E . Diet quality and risk of frailty among older women in the Nurses’ Health Study. Am J Clin Nutr. 2020;111(4):877‐883. 32091575 10.1093/ajcn/nqaa028 PMC7138663

[6] Soysal P , Stubbs B , Lucato P , et al. Inflammation and frailty in the elderly: a systematic review and meta‐analysis. Ageing Res Rev. 2016;31:1‐8. 27592340 10.1016/j.arr.2016.08.006

[7] Anand A , Cudmore S , Robertson S , et al. Frailty assessment and risk prediction by GRACE score in older patients with acute myocardial infarction. BMC Geriatr. 2020;20(1):102. 32164580 10.1186/s12877-020-1500-9 PMC7069195

[8] Mauthner O , Claes V , Deschodt M , et al. Handle with care: a systematic review on frailty in cardiac care and its usefulness in heart transplantation. Transplant Rev. 2017;31(3):218‐224. 10.1016/j.trre.2017.03.003 28390796

[9] Sze S , Pellicori P , Zhang J , Weston J , Clark AL . Identification of frailty in chronic heart failure. JACC Heart Fail. 2019;7(4):291‐302. 30738977 10.1016/j.jchf.2018.11.017

[10] Stang A . Critical evaluation of the Newcastle‐Ottawa scale for the assessment of the quality of nonrandomized studies in meta‐analyses. Eur J Epidemiol. 2010;25(9):603‐605. 20652370 10.1007/s10654-010-9491-z

[11] Higgins JP , Thompson SG , Deeks JJ , Altman DG . Measuring inconsistency in meta‐analyses. BMJ. 2003;327(7414):557‐560. 12958120 10.1136/bmj.327.7414.557 PMC192859

[12] Sterne JA , Egger M . Funnel plots for detecting bias in meta‐analysis: guidelines on choice of axis. J Clin Epidemiol. 2001;54(10):1046‐1055. 11576817 10.1016/s0895-4356(01)00377-8

[13] Uchmanowicz I , Lisiak M , Wleklik M , Gurowiec P , Kałużna‐Oleksy M . The relationship between frailty syndrome and quality of life in older patients following acute coronary syndrome. Clin Interv Aging. 2019;14:805‐816. 31190767 10.2147/CIA.S204121 PMC6511650

[14] Dodson JA , Hochman JS , Roe MT , et al. The association of frailty with in‐hospital bleeding among older adults with acute myocardial infarction: insights from the ACTION registry. JACC Cardiovasc Interv. 2018;11(22):2287‐2296. 30466828 10.1016/j.jcin.2018.08.028 PMC6260951

[15] Patel A , Goodman SG , Yan AT , et al. Frailty and outcomes after myocardial infarction: insights from the CONCORDANCE registry. J Am Heart Assoc. 2018;7(18):e009859. 30371219 10.1161/JAHA.118.009859 PMC6222944

[16] Nguyen TV , Le D , Tran KD , Bui KX , Nguyen TN . Frailty in older patients with acute coronary syndrome in Vietnam. Clin Interv Aging. 2019;14:2213‐2222. 31908432 10.2147/CIA.S234597 PMC6925543

[17] Yoshioka N , Takagi K , Morishima I , et al. Influence of preadmission frailty on short‐ and mid‐term prognoses in octogenarians with ST‐elevation myocardial infarction. Circ J. 2019;84(1):109‐118. 31787661 10.1253/circj.CJ-19-0467

[18] Hermans MPJ , Eindhoven DC , van Winden LAM , et al. Frailty score for elderly patients is associated with short‐term clinical outcomes in patients with ST‐segment elevated myocardial infarction treated with primary percutaneous coronary intervention. Neth Heart J. 2019;27(3):127‐133. 30771094 10.1007/s12471-019-1240-7 PMC6393578

[19] Nishihira K , Yoshioka G , Kuriyama N , et al. Impact of frailty on outcomes in elderly patients with acute myocardial infarction who undergo percutaneous coronary intervention. Eur Heart J Qual Care Clin Outcomes. 2021;7(2):189‐197. 32142106 10.1093/ehjqcco/qcaa018

[20] Calvo E , Teruel L , Rosenfeld L , et al. Frailty in elderly patients undergoing primary percutaneous coronary intervention. Eur J Cardiovasc Nurs. 2019;18(2):132‐139. 30156426 10.1177/1474515118796836

[21] Afilalo J , Alexander KP , Mack MJ , et al. Frailty assessment in the cardiovascular care of older adults. J Am Coll Cardiol. 2014;63(8):747‐762. 24291279 10.1016/j.jacc.2013.09.070 PMC4571179

[22] Rockwood K , Song X , MacKnight C , et al. A global clinical measure of fitness and frailty in elderly people. CMAJ. 2005;173(5):489‐495. 16129869 10.1503/cmaj.050051 PMC1188185

[23] Puymirat E , Simon T , Steg PG , et al. Association of changes in clinical characteristics and management with improvement in survival among patients with ST‐elevation myocardial infarction. JAMA. 2012;308(10):998‐1006. 22928184 10.1001/2012.jama.11348

[24] Sujino Y , Tanno J , Nakano S , et al. Impact of hypoalbuminemia, frailty, and body mass index on early prognosis in older patients (≥85 years) with ST‐elevation myocardial infarction. J Cardiol. 2015;66(3):263‐268. 25547740 10.1016/j.jjcc.2014.12.001

[25] Qin Y , Wei X , Han H , et al. Association between age and readmission after percutaneous coronary intervention for acute myocardial infarction. Heart. 2020;106(20):1595‐1603. 32144190 10.1136/heartjnl-2019-316103

[26] Manoukian SV , Feit F , Mehran R , et al. Impact of major bleeding on 30‐day mortality and clinical outcomes in patients with acute coronary syndromes: an analysis from the ACUITY trial. J Am Coll Cardiol. 2007;49(12):1362‐1368. 17394970 10.1016/j.jacc.2007.02.027

[27] Alexander KP , Chen AY , Roe MT , et al. Excess dosing of antiplatelet and antithrombin agents in the treatment of non‐ST‐segment elevation acute coronary syndromes. JAMA. 2005;294(24):3108‐3116. 16380591 10.1001/jama.294.24.3108

[28] Bromage DI , Jones DA , Rathod KS , et al. Outcome of 1051 octogenarian patients with ST‐segment elevation myocardial infarction treated with primary percutaneous coronary intervention: observational cohort from the London Heart Attack Group. J Am Heart Assoc. 2016;5(6):e003027. 27353606 10.1161/JAHA.115.003027 PMC4937253

[29] Tsao CW , Aday AW , Almarzooq ZI , et al. Heart disease and stroke statistics—2022 update: a report from the American Heart Association. Circulation. 2022;145(8):e153‐e639. 35078371 10.1161/CIR.0000000000001052

[30] Smith JR , Thomas RJ , Bonikowske AR , Hammer SM , Olson TP . Sex differences in cardiac rehabilitation outcomes. Circ Res. 2022;130(4):552‐565. 35175838 10.1161/CIRCRESAHA.121.319894 PMC8868533

[31] Baman JR , Sekhon S , Maganti K . Cardiac rehabilitation. JAMA. 2021;326(4):366. 34313685 10.1001/jama.2021.5952

[32] Ibanez B , James S , Agewall S , et al. 2017 ESC guidelines for the management of acute myocardial infarction in patients presenting with ST‐segment elevation: The Task Force for the management of acute myocardial infarction in patients presenting with ST‐segment elevation of the European Society of Cardiology (ESC). Eur Heart J. 2018;39(2):119‐177. 28886621 10.1093/eurheartj/ehx393

[33] Heran BS , Chen JM , Ebrahim S , et al. Exercise‐based cardiac rehabilitation for coronary heart disease. Cochrane Database Syst Rev. 2011;7:CD001800. 10.1002/14651858.CD001800.pub2 PMC4229995 21735386

[34] McMahon SR , Ades PA , Thompson PD . The role of cardiac rehabilitation in patients with heart disease. Trends Cardiovasc Med. 2017;27(6):420‐425. 28318815 10.1016/j.tcm.2017.02.005 PMC5643011

