# Supplementary material for: Prevalence and adverse outcomes of frailty in older patients with acute myocardial infarction after percutaneous coronary interventions: A systematic review and meta‐analysis
Source: Clin Cardiol. 2022 Sep 28;46(1):5–12. doi: 10.1002/clc.23929 (PMC9849439; doi:10.1002/clc.23929)
Supplement: Supplementary file 1 — Supplementary information. [file CLC-46-5-s002.pdf]

**Supplement Table 1 Subgroup analyses of the prevalence of AMI after PCI in frail older participants**

| | Fixed-effect model | | | Random-effect model | | | | | | |
|---|---|---|---|---|---|---|---|---|---|---|
| analysis | Prevalence | 95%CI | P(z-text) | Prevalence | 95%CI | P(z-text) | I-squared(%) | P | Model | *P-values for heterogeneity from meta-regression |
| **Study design** | | | | | | | | | | |
| Cross-sectional study and cohort study | 0.10 | (0.10-0.10) | 0.000 | 0.36 | (0.19-0.52) | 0.000 | NA | 0.000 | Random | 0.565 |
| Cross-sectional study | 0.48 | (0.48-0.48) | 0.000 | 0.59 | (0.12-1.06) | 0.013 | 100.00 | 0.000 | Random | |
| Cohort study | 0.28 | (0.22-0.34) | 0.000 | 0.28 | (0.22-0.34) | 0.000 | | | Random | |
| Prospective study | 0.25 | (0.22-0.28) | 0.000 | 0.24 | (0.16-0.32) | 0.000 | 85.10 | 0.010 | Random | |
| **Sex%** | | | | | | | | | | |
| Male | 0.56 | (0.55-0.56) | 0.000 | 0.55 | (0.50-0.61) | 0.000 | 99.90 | 0.000 | Random | 1.000 |
| Female | 0.44 | (0.44-0.45) | 0.000 | 0.45 | (0.39-0.50) | 0.000 | 99.90 | 0.000 | Random | |
| **Sample size** | | | | | | | | | | |
| <500 | 0.38 | (0.35-0.41) | 0.000 | 0.43 | (0.24-0.63) | 0.000 | 97.90 | 0.000 | Random | 0.070 |
| ≥500 | 0.29 | (0.29-0.29) | 0.000 | 0.36 | (0.07-0.64) | 0.013 | 100.00 | 0.000 | Random | |
| **Frailty assessment tool** | | | | | | | | | | |
| Frailty index | 0.10 | (0.10-0.10) | 0.000 | 0.29 | (0.09-0.50) | 0.005 | 99.80 | 0.000 | Random | 0.459 |
| Frailty Point Scoring System | 0.48 | (0.48-0.48) | 0.000 | 0.49 | (-0.08-1.06) | 0.093 | 100.00 | 0.000 | Random | |
| Others | 0.34 | (0.32-0.37) | 0.000 | 0.41 | (0.26-0.56) | 0.000 | 97.60 | 0.000 | Random | |
| **Populations** | | | | | | | | | | |
| STEMI undergone PCI | 0.78 | (0.78-0.78) | 0.000 | 0.45 | (0.22-0.69) | 0.000 | 99.70 | 0.000 | Random | 0.304 |
| NSTEMI undergone PCI | 0.20 | (0.20-0.20) | 0.000 | 0.40 | (0.29-0.51) | 0.000 | 99.30 | 0.000 | Random | |
| AMI undergone PCI | 0.10 | (0.10-0.10) | 0.000 | 0.26 | (0.09-0.43) | 0.003 | 98.70 | 0.000 | Random | |
| **Quality** | | | | | | | | | | |
| Medium quality | 0.36 | (0.32-0.40) | 0.000 | 0.50 | (-0.09-1.09) | 0.099 | 99.40 | 0.000 | Random | 0.383 |
| High quality | 0.29 | (0.29-0.29) | 0.000 | 0.37 | (0.14-0.60) | 0.002 | 100.00 | 0.000 | Random | |
