# Supplementary material for: Prevalence and adverse outcomes of frailty in older patients with acute myocardial infarction after percutaneous coronary interventions: A systematic review and meta‐analysis
Source: Clin Cardiol. 2022 Sep 28;46(1):5–12. doi: 10.1002/clc.23929 (PMC9849439; doi:10.1002/clc.23929)
Supplement: Supplementary file 2 — Supplementary information. [file CLC-46-5-s003.pdf]

Supplement Table 2 Subgroup analyses of adverse outcomes in frail older participants with AMI after PCI

	Fixed-effect model			Random-effect model						
**Analysis**	**HR**	**95%CI**	**P(z-text)**	**HR**	**95%CI**	**P(z-text)**	**I-squared(%)**	**P**	**Model**	**P-values for heterogeneity from meta-regression**
**Study design**										
Cohort study	2.21	(1.25-3.58)	0.005	2.42	(1.12-5.22)	0.025	36.20	0.209	Fixed	
Prospective study	1.92	(1.45-2.54)	0.000	1.92	(1.45-2.54)	0.000	0.00	0.490	Fixed	0.225
Cross-sectional study，cohort study	2.69	(1.81-3.99)	0.000	2.69	(1.81-3.99)	0.000	0.00	0.804	Fixed	
**Follow-up**										
<1years	3.04	(1.94-4.77)	0.000	3.04	(1.94-4.77)	0.000	0.00	0.370	Fixed	0.118
≥1years	1.89	(1.49-2.41)	0.000	1.89	(1.49-2.41)	0.000	0.00	0.972	Fixed	
N/A	3.96	(1.16-13.54)	0.028	3.96	(1.16-13.54)	0.028			Fixed	
**Sample size**										
<500	2.54	(1.87-3.45)	0.000	2.54	(1.87-3.45)	0.000	0.00	0.594	Fixed	0.183
≥500	1.85	(1.39-2.46)	0.000	1.85	(1.39-2.46)	0.000	0.00	0.869	Fixed	
**Frailty assessment tool**										
Frailty Point Scoring System	1.85	(1.43-2.38)	0.000	1.85	(1.43-2.38)	0.000	0.00	0.986	Fixed	0.082
Reported Edmonton Frail Scale	2.82	(1.77-4.48)	0.000	2.82	(1.77-4.48)	0.000	0.00	0.590	Fixed	
Others	3.16	(1.72-5.79)	0.000	3.24	(1.69-6.22)	0.000	7.90	0.338	Fixed	
**Population**										
AMI undergone PCI	2.03	(1.43-2.38)	0.000	2.03	(1.63-2.54)	0.000	0.00	0.719	Fixed	0.225
STEMI undergone PCI	3.16	(1.72-5.79)	0.000	3.24	(1.69-6.22)	0.000	7.90	0.338	Fixed	
**Type**										
STEMI undergone PCI	3.16	(1.72-5.79)	0.000	3.24	(1.69-6.22)	0.000	7.90	0.338	Fixed	0.225
Unclear	2.03	(1.63-2.54)	0.000	2.03	(1.63-2.54)	0.000	0.00	0.719	Fixed	
**Quality**										
Medium quality	2.08	(1.26-3.44)	0.004	2.08	(1.26-3.44)	0.004	0.00	0.504	Fixed	0.906
High quality	2.16	(1.71-2.72)	0.000	2.18	(1.74-2.79)	0.000	0.08	0.365	Fixed	
