# Supplementary figures and images for: Prevalence and adverse outcomes of frailty in older patients with acute myocardial infarction after percutaneous coronary interventions: A systematic review and meta‐analysis
Source: Clin Cardiol. 2022 Sep 28;46(1):5–12. doi: 10.1002/clc.23929 (PMC9849439; doi:10.1002/clc.23929)

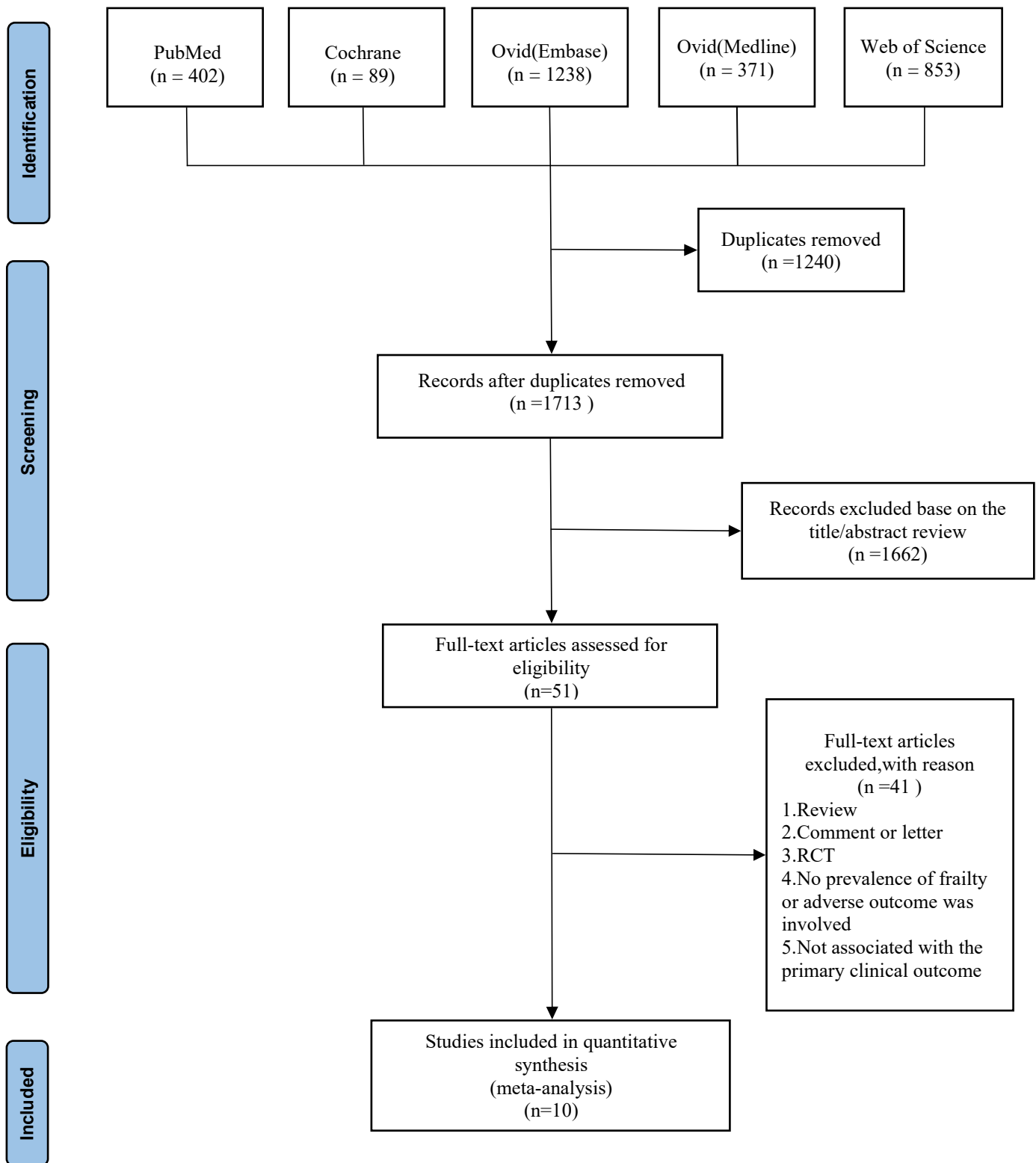

Supplement Figure 1 The flow diagram of studies selection

Supplement: Supplementary file 3 — Supplementary information. [file CLC-46-5-s001.pdf]
